# TEMPORAL TRENDS IN PANCREATIC CANCER MORTALITY IN BRAZIL BETWEEN 1980 AND 2023: ARE WE ALIGNED WITH THE GLOBAL PROJECTIONS?

**DOI:** 10.1590/S0004-2803.24612025-070

**Published:** 2026-07-20

**Authors:** Lucas Casagrande Passoni LOPES, Isabela Ussifati NEGRINE, Carlos Antonio NEGRATO

**Affiliations:** * Universidade de São Paulo, Faculdade de Medicina de Bauru, Bauru, São Paulo , Brazil.

**Keywords:** Pancreatic cancer, diabetes mellitus, Brazil, sexes, Câncer de pâncreas, diabetes mellitus, Brasil, sexo

## Abstract

**Background:**

Pancreatic cancer (PC) is a highly lethal malignancy with rising incidence and mortality rates, although its burden remains poorly investigated in Brazil. Understanding its regional and sex-specific trends is critical for tailoring public health interventions and mitigating the disease’s impact in the Brazilian population.

**Objective:**

This study aimed to evaluate the temporal trends in PC mortality among Brazilian individuals between 1980 and 2023 and its alignment with the global projections.

**Methods:**

This retrospective, population-based ecological study analyzed PC mortality data using the Sistema de Informação de Mortalidade (SIM) and demographic data from the Instituto Brasileiro de Geografia e Estatística (IBGE). Annual Percent Change (APC) calculations were performed to assess temporal trends by Brazilian geographic regions and sex, accounting for demographic shifts over time.

**Results:**

A total of 257,671 PC-related deaths occurred during the study period, averaging 5,856 deaths per year. The overall mortality trend for PC in Brazil showed a continuous increase of (APC: 1.23; 95%CI: 1.16-1.32; *P*-value <0.01). Regional analyses revealed significant increases in the North (APC: 2.32), South (APC: 0.59), and Midwest (APC: 1.45) regions. Sex-specific trends indicated a steady increase for women throughout the period, while men experienced alternating phases of rising and stationary trends.

**Conclusion:**

PC mortality in Brazil has risen significantly over the past four decades, with marked regional and sex-specific disparities, aligning with global perspectives. These findings highlight the need for targeted PC prevention, early detection, and equitable access to high-quality cancer care, particularly in vulnerable regions and populations.

## HIGHLIGHTS

• Pancreatic cancer mortality in Brazil showed a continuous increase between 1980 and 2023.

• A total of 257,671 deaths were recorded during this period, with an annual average of 5,856 deaths.

• The North, South, and Central-West regions showed significant upward trends in mortality.

• A continuous increase in mortality was observed among women, while men showed alternating phases of growth and stability.

• The results reflect global trends and reinforce the need for targeted strategies for prevention, early diagnosis, and equitable access to treatment.

## Resumo

**Contexto:**

O câncer de pâncreas (CP) é uma neoplasia altamente letal, com incidência e mortalidade crescentes em todo o mundo. Apesar de seu impacto significativo, a carga da doença permanece pouco investigada no Brasil, especialmente quanto às diferenças regionais e entre sexos, aspectos fundamentais para orientar estratégias de saúde pública.

**Objetivo:**

Avaliar as tendências temporais da mortalidade por câncer de pâncreas no Brasil entre 1980 e 2023 e verificar seu alinhamento com as projeções epidemiológicas globais.

**Métodos:**

Estudo ecológico retrospectivo de base populacional utilizando dados de mortalidade do Sistema de Informação sobre Mortalidade (SIM) e estimativas populacionais do Instituto Brasileiro de Geografia e Estatística (IBGE). Os óbitos por câncer de pâncreas foram identificados pelos códigos ‘157 da CID-9 e C25 da CID-10. As tendências temporais foram avaliadas por meio do cálculo da Variação Percentual Anual (Annual Percent Change - APC) utilizando análise de joinpoint, estratificada por sexo e regiões geográficas do Brasil.

**Resultados:**

Entre 1980 e 2023 foram registrados 257.671 óbitos por câncer de pâncreas no Brasil, com média anual de 5.856 mortes. Observou-se tendência global crescente de mortalidade ao longo de todo o período analisado (APC: 1,23; IC95%: 1,16-1,32; p<0,01). Nas análises regionais, foram identificadas tendências significativas de aumento nas regiões Norte (APC: 2,32), Sul (APC: 0,59) e Centro-Oeste (APC: 1,45). Entre os sexos, as mulheres apresentaram aumento contínuo da mortalidade ao longo de todo o período, enquanto os homens apresentaram fases alternadas de crescimento e estabilidade nas taxas de mortalidade.

**Conclusão:**

A mortalidade por câncer de pâncreas no Brasil aumentou significativamente nas últimas quatro décadas, com importantes disparidades regionais e diferenças entre os sexos. Esses achados acompanham as tendências observadas globalmente e destacam a necessidade de estratégias direcionadas de prevenção, diagnóstico precoce e ampliação do acesso equitativo ao cuidado oncológico, especialmente em regiões e populações mais vulneráveis.

## INTRODUCTION

Pancreatic cancer (PC) is a highly aggressive malignant neoplasm originating from the exocrine or endocrine tissues of the pancreas. The vast majority of cases (approximately 90%) are pancreatic ductal adenocarcinomas, which arise from the epithelial cells lining the pancreatic ducts. Less common histological subtypes include acinar cell carcinoma, neuroendocrine tumors, and the rare pancreatoblastoma, each one presenting distinct biological behaviors and clinical outcomes^1^.

Despite accounting for only about 3% of all cancers globally, PC is an important cause of cancer-related mortality due to its aggressive nature and poor prognosis. Overall, PC affects predominantly older adults, being rare before the age of 40 and most commonly found after the age of 60^2^ ^,^ ^3^. Globally, it ranks as the seventh leading cause of cancer death, with an estimated 495,773 new cases and 466,003 deaths reported in 2020^4^ ^,^ ^7^. Its incidence has been rising steadily, particularly in high-income countries, likely reflecting shifts in known risk factors such as smoking, obesity, diabetes, and populations ageing. Notably, recent projections suggest that PC will become the second leading cause of cancer death in the European Union by 2025 and the third in the United States by 2030, underscoring the urgent need for improved early detection and treatment strategies^4^.

In Brazil, PC accounts for approximately 1% of all cancer diagnoses but contributes to around 5% of all cancer-related deaths, reflecting its disproportionately high lethality^5^. However, despite its significant impact, comprehensive data on its epidemiological profile remain scarce. This gap in knowledge is particularly concerning given the increasing prevalence of risk factors such as obesity and diabetes in the country, which may further drive PC incidence in the coming decades. Without a clear understanding of local epidemiological patterns, it is challenging to develop effective public health strategies or allocate healthcare resources appropriately.

Therefore, this study aimed to assess PC mortality rates in Brazil from 1980 to 2023, with the goal of determining whether the country’s epidemiological patterns mirror the alarming global trends and to identify potential areas for intervention and health policy improvement.

## METHODS

### Study design

This was a retrospective, population-based study that evaluated temporal trends in PC mortality rates in Brazil between 1980 and 2023, in order to determine if Brazil is aligned with the global projections.

### Study location

The study was located in Brazil, the largest country in South America and the fifth in the world. Its territory is divided into 27 states and a Federal District, which are distributed in five geographic macroregions: North, Northeast, Midwest, Southeast and South. 

Each one of them presents a unique blend of cultural identity and economic activities. For instance, the North region is marked by the Amazon rainforest and agro-extractive activities. In parallel, the Southeast leads the country’s economy, characterized as the most urbanized and industrialized region. Beyond these differences, the existing regional disparities in development underscore Brazil’s diversity and its challenges in achieving equitable growth that may impact a lot of health issues, such as PC-related deaths, for instance.

### Data sources

All data were extracted from DATASUS, an online and open-access platform that houses Brazilian public health and demographic data and can be accessed in the following link: https://datasus.saude.gov.br/.

The study period, spanning from 1980 to 2023, was selected since it represents the timeframe for which comprehensive and consistent data were available in the utilized database. Mortality records were obtained from the Sistema de Informação de Mortalidade (SIM), a national public health information system maintained by the Brazilian Ministry of Health. This system compiles all registered deaths occurring within the country, providing a reliable source of mortality data.

For this analysis, deaths attributed to PC were identified using the International Classification of Diseases, Ninth Revision (ICD-9) code 157 and the corresponding Tenth Revision (ICD-10) code C25, which together encompass all PC-related deaths. Mortality data were extracted for males, females, and the total population across each of the five major geographic regions of Brazil, as defined by the Instituto Brasileiro de Geografia e Estatística (IBGE), the national authority responsible for census and demographic statistics.

Importantly, more granular data on PC mortality, such as stratifications by ethnicity, comorbidities, specific PC treatment modalities, and individual age groups, were not included in the analysis, as this information was not available in the utilized dataset.

Population estimates, including the total number of inhabitants, as well as the male and female populations in each of these geographic regions from 1980 to 2023, were also obtained from the IBGE. These demographic data were essential for accurately calculating mortality rates and adjusting for population changes over time.

### Statistical analysis

In this study, the Annual Percent Change (APC) was calculated to quantify temporal trends in PC mortality over the specified period. This approach accounts for shifts in both population size and age structure, allowing for a more precise assessment of mortality trends over time. The APC calculation was performed separately for each of the five major geographic regions and for both sexes, ensuring that regional and sex-specific variations were appropriately captured.

Statistically, a positive APC indicates a rising trend in mortality rates, suggesting that the number of PC-related deaths has been increasing over time. Conversely, a negative APC reflects a declining trend, indicating that PC mortality rates have decreased. If the calculated 95% confidence interval (CI) for the APC does not include zero, the trend is considered statistically significant (*P*-value <0.05), confirming that the observed change is unlikely to have occurred by chance. On the other hand, if the CI includes zero (*P*-value ≥0.05), the trend is considered stationary, implying no significant increase or decrease in mortality over the assessed period. 

The analysis was performed using the joinpoint software 5.4® and the R software 4.4.3®.

## RESULTS

**FIGURE 1:**
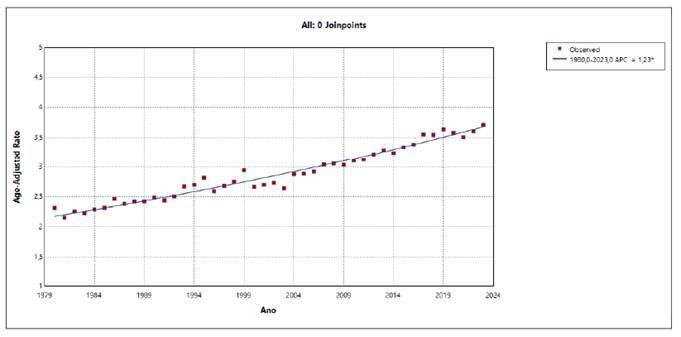
Temporal trends in pancreatic cancer mortality in Brazil between 1980 and 2023.

Over the evaluated period, a total of 257,671 deaths by PC occurred in Brazil, which means an average of 5,856.16 deaths by year. The joinpoint analysis revealed an increasing mortality trend over all the evaluated period with no inflexion points (APC: 1.23; 95%CI: 1.16 - 1.32; *P*-value <0.01), as can be seen in Figure 1. Table 1 shows the main results obtained.

**TABLE 1:** Summary of the main findings.

	Total deaths	Annual average deaths	Period	Joinpoint analysis	Meaning
Brazil	257,671	5,856.16	1980-2023	1.23; 1.16-1.32; <0.01	↑
Sex					
Men	130,466	2,965.13	1980-1998	0.92; 0.57-3.22; 0.02	↑
			1999-2001	1.67; -2.94-2.28; 0.39	↔
			2002-2017	1.81; 0.14-3.81; 0.04	↑
			2018-2023	0.24; -1.67-1.44; 0.64	↔
Women	127,205	2,891.02	1980-1997	1.95; 1.57-7.11; <0.01	↑
			1998-2023	1.33; 0.57-1.48; 0.03	↑
Geographic regions					
North	8,189	186.11	1980-2023	2.32; 2.06-2.79; <0.01	↑
Midwest	14,557	330.84	1980-2023	1.45; 1.23-1.80; <0.01	↑
South	58,004	1,318.27	1980-2023	0.59; 0.50-0.72; <0.01	↑
Northeast	41,843	950.97	1980-1999	1.55; 0.59-2.29; <0.01	↑
			2000-2006	7.40; 5.32-13.92; <0.01	↑
			2007-2023	2.49; 1.96-2.89; <0.01	↑
Southeast	135,078	3,069.95	1980-1998	1.17; 0.90-1.78; <0.01	↑
			1999-2002	-1.98; -3.87-0.13; 0.07	↔
			2003-2023	1.07; 0.89-1.37; <0.01	↑

APC; 95%CI; *P*-value. ↑, Increasing trend. ↔, stationary trend.

**FIGURE 2:**
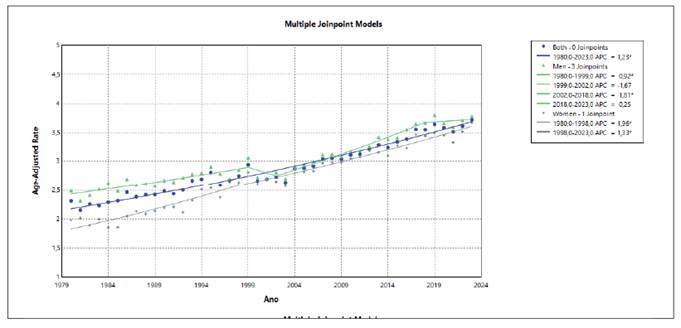
Temporal trends in pancreatic cancer mortality in Brazil between 1980 and 2023 according to sex.

Stratifying the analysis by sex, a total of 130,466 PC-related deaths occurred in men and 127,205 in women over the period, which represents an average of 2,965.13 and 2,891.02 deaths yearly for men and women, respectively. Regarding male gender, between 1980 and 1998, an increasing mortality trend (APC: 0.92; 95%CI: 0.57 - 3.22; *P*-value: 0.02) was observed. Between 1999 and 2001, a stationary mortality trend was found (APC: - 1.67; 95%CI: -2.94 - 2.28; *P*-value: 0.39). Between 2002 and 2017, an increasing trend (APC: 1.81; 95%CI: 0.14 - 3.81; *P*-value: 0.04) was observed and between 2018 and 2023, a stationary trend was found again (APC: 0.24; 95%CI: -1.67 - 1.44; *P*-value: 0.64). Among 1980 and 1997, women presented an increasing trend (APC: 1.95; 95%CI: 1.57 - 7.11; *P*-value <0.01). Betwixt 1998 and 2023, mortality trends in women continued to increase, but less pronounced than in the previous years (APC: 1.33; 95%CI: 0.57 - 1.48; *P*-value: 0.03) - Figure 2.

**FIGURE 3:**
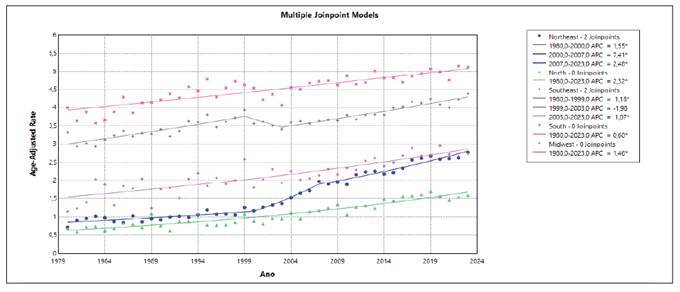
Temporal trends in pancreatic cancer mortality in Brazil between 1980 and 2023 according to Brazilian geographic region.

**FIGURE 4:**
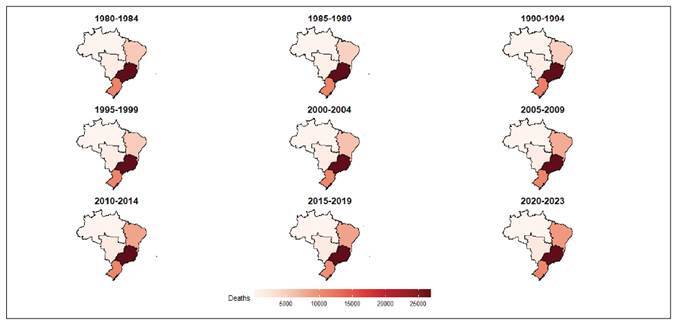
Heatmap showing temporal trends in pancreatic cancer mortality in Brazil between 1980 and 2023 according to Brazilian geographic region.

As for the Brazilian regions, it was observed that a total of 41,843, 8,189, 135,078, 58,004 and 14,557 deaths, occurred in Northeast, North, Southeast, South and Midwest regions, respectively. This corresponds to an annual average number of deaths of 950.97, 186.11, 3,069.95, 1,318.27 and 330.84 for the Northeast, North, Southeast, South and Midwest regions, respectively. Throughout the evaluated period, it could be observed that the North (APC: 2.32; 95%CI: 2.06 - 2.79; *P*-value <0.01), South (APC: 0.59; 95%CI: 0.50 - 0.72; *P*-value <0.01) and Midwest (APC: 1.45; 95%CI: 1.23 - 1.80; *P*-value <0.01) regions presented a continuous increasing trend in mortality due to PC, without any inflection point. The Northeast region presented increasing trends in mortality throughout the period. Between 1980 and 1999 (APC: 1.55; 95%CI: 0.59 - 2.29; *P*-value <0.01), between 2000 and 2006 (APC: 7.40; 95%CI: 5.32 - 13.92; *P*-value <0.01) and between 2007 and 2023 (APC: 2.49; 95%CI: 1.96 - 2.89; *P*-value <0.01). Finally, the southeast region showed an increasing mortality trend between 1980 and 1998 (APC: 1.17; 95%CI: 0.90 - 1.78; *P*-value <0.01), a stationary trend between 199 and 2002 (APC: -1.98; 95%CI: -3.87 - 0.13; *P*-value: 0.07) and a new increasing trend between 2003 and 2023 (APC: 1.07; 95%CI: 0.89 - 1.37; *P*-value <0.01) - Figure 3 and 4.

## DISCUSSION

The present study highlights an overall 1.23% increased trend in PC-related mortality, between 1980 and 2023, with no inflection points. When stratified by sex, both men and women exhibited increasing trends; however, the trajectory was more oscillating in men, with alternating periods of increasing and stationarity trends. Locally, all Brazilian regions showed rising mortality trends, with the North, South, and Midwest experiencing uninterrupted increases. The Northeast exhibited sharp peaks, particularly from 2000 to 2006, while the Southeast displayed a brief period of stabilization between 1999 and 2002 before resuming an upward trend.

Our findings show a particularly concerning trend: in Brazil, PC mortality increased at an annual rate of 1.23%. Globally, PC incidence is projected to be next to 19 cases for 100,000 individuals by 2050, which represents an annual increase of more than 1%^4^. Some countries have documented even more dramatic increases. For example, a study conducted with data of different countries reported increasing PC incidence in some European countries with highlights to Iceland, Cyprus and France, which demonstrated the most relevant trends^5^. Similarly, a study reported that PC mortality are increasing with annual rates of about 10% in Turkmenistan^6^. In Eastern Europe, some countries such as Hungary, Slovakia, and the Czech Republic stand out for having high incidence and mortality rates from PC, with these rates predicted to continue increasing until 2040^7^.

The rising global mortality rates associated with PC can be attributed to a multifaceted interplay of demographic, biological, and healthcare-related factors^8^ ^-^ ^10^. The aging of populations is a fundamental driver, as the incidence of PC increases significantly with age, and life expectancy has steadily risen worldwide^8^. Additionally, the disease’s asymptomatic early stages and the absence of effective screening programs result in late-stage diagnoses, limiting curative treatment options^9^. The growing prevalence of key risk factors-such as obesity, smoking, and chronic pancreatitis-further exacerbates the disease burden by inducing metabolic and inflammatory changes that can promote carcinogenesis^9^. Furthermore, shifts in environmental exposures and lifestyle behaviors, including increased consumption of processed and ultra-processed foods, high-fat diets, and sedentary lifestyle, may contribute to the rising incidence of PC^10^.

Conflicting data exist regarding sex impact on PC mortality^11^ ^-^ ^13^. An evaluation from the Global Burden of Diseases Study pointed out that men tend to present higher mortality rates than women^11^. Possibly, biological mechanisms, such as androgen receptor signaling and estrogen-mediated anti-inflammatory effects, may explain some of these differences^11^. Additionally, a study observed that men tend to smoke more than women. However, PC mortality risk is close in both sexes, with both of them being benefited from ceasing such a habit^12^. In parallel, a Brazilian study has found that PC mortality trends have increased more importantly in women in comparison with men, with the authors discussing the impact of differential epidemiological and demographic transitional elements on explaining these and their other findings^13^.

In Brazil, the North and Midwest regions, which have shown continuously increasing mortality trends, historically face significant healthcare accessibility challenges^14^ ^,^ ^15^. Comparatively, in rural communities of the United States slight increasing trends in PC mortality were also observed, possibly driven by limited healthcare access that leads to later-stage diagnoses and reduced survival rates^14^. The sharp increase in PC mortality in the Northeast, as well as the one observed in large urban centers like Shanghai, in China, may be explained by a rapid process of industrialization and ubranization, which changed the socioeconomic life of its inhabitants^15^. The Southeast region, the richest in Brazil, has also experienced increasing PC mortality rates. This is in accordance with data found in highly developed European nations in which PC continues to drive mortality rates upward^5.6^. These comparisons underscore the multifaceted nature of PC mortality disparities, influenced by healthcare accessibility, economic development, and population health dynamics^16^. Addressing these disparities requires regionally specific public health interventions that improve early detection, enhance healthcare accessibility, and mitigate metabolic risk factors on a global scale^16^.

In the present study, a significant divergence in trends was observed among different Brazilian regions. This aligns with a series of previous studies that have also observed similar disparities^17^ ^-^ ^20^. In this sense, a study conducted with data from the global Burden of Diseases noted trends of increasing mortality from PC in Brazil as a whole^17^. However, it was observed that the trends from the North and Northeast regions of the country showed an even more significant increase, which could be associated with lower sociodemographic indexes levels in these locations^17^. In parallel, another Brazilian study conducted with national data observed that the highest mortality rates from PC were noted in regions with higher human development indices^18^. This could be explained by better healthcare coverage in these areas, leading to more diagnoses of this condition and a better level of recognition and notification of its occurrence^18^. Interestingly, other studies have observed that mortality rates are not only linked to social and economic issues, but also take into account population size and density, age structure, the prevalence of risk factors such as alcohol and tobacco consumption, and even immigration and emigration as adjustment factors to be considered in interpreting the topic^19^ ^,^ ^20^.

We were unable to access data regarding patients’ age at death, ethnicity, and the existence of comorbidities such as Type 2 diabetes mellitus (T2MD), which might significantly impact PC mortality^21^ ^,^ ^22^. Ethnicity plays a crucial role in mortality disparities, in which Black individuals exhibit higher Age-Standardized Mortality Rate (ASMR) compared to White people^21^. These disparities may result from variations in healthcare access, socioeconomic factors, and potential biological differences^21^. Age is another key determinant of PC mortality, with individuals aged 65 and older experiencing the highest mortality rates^22^. Advanced age is associated with worse survival prognosis, with individuals over 80 years old facing a threefold higher mortality risk compared to those under 40^22^. It should also be mentioned that long-standing T2DM is recognized as a significant risk factor for PC^23^.

Chronic hyperglycemia, insulin resistance, and compensatory hyperinsulinemia create a pro-tumorigenic environment by promoting cellular proliferation, inhibiting apoptosis, and enhancing inflammatory pathways that contribute to pancreatic carcinogenesis^24^ ^,^ ^25^. Conversely, T2DM itself can also be an early manifestation of PC, often preceding the diagnosis by months or even years^24^. This occurs due to tumor-induced beta-cell dysfunction and altered glucose metabolism, leading to new-onset T2DM in a subset of patients^24^. Given the high global prevalence of T2DM, even a moderate increase in relative risk translates into a substantial contribution to the overall burden of PC, reinforcing the importance of identifying high-risk individuals for as early as possible detection and intervention^25^.

Understanding the temporal trends in PC mortality, as revealed by this study, is crucial for guiding public health interventions and resource allocation^26^. The observed regional disparities and sex-specific differences underscore the need for tailored strategies in cancer prevention and early detection^26^. Policymakers can utilize these insights to prioritize regions with the steepest mortality increases, such as the North and Northeast, and focus on reducing known risk factors, enhancing diagnostic capacities, and ensuring equitable access to treatment^26^. This information is also essential for refining national cancer control plans and targeting high-risk populations, ultimately reducing the overall burden of PC in Brazil^26^.

This study, while comprehensive, does have some limitations. The analysis relied exclusively on aggregate mortality data from SIM ad IBGE, which do not provide detailed patient-level information. This prevented the evaluation of key factors such as ethnicity, comorbidities, specific PC treatments, and age distributions, all of which could significantly influence mortality outcomes. Additionally, potential inaccuracies in death certification and regional disparities in healthcare infrastructure might have introduced biases in the reported mortality rates.

Despite these limitations, this study presents significant strengths. It is the first to provide a long-term, population-based analysis of PC mortality trends across all five Brazilian regions, spanning over four decades. The use of standardized classification systems (ICD-9 and ICD-10) ensures comparability with international data, while the incorporation of APC analyses allows for precise identification of temporal shifts in mortality trends. The extensive timeframe and comprehensive geographic coverage provide a robust foundation for understanding regional disparities and guiding future public health strategies.

## CONCLUSION

This study demonstrated that PC mortality in Brazil has increased over the past four decades, with distinct regional and sex-specific variations, in alignment with the global projections. Public health policymakers should strengthen PC prevention, expanding early detection programs, and ensuring equitable access to high-quality cancer care.

## Data Availability

Research data are available in the public database DATASUS (https://datasus.saude.gov.br/).
